# Complete Genome Sequence of the Nonylphenol-Degrading Bacterium *Sphingobium cloacae* JCM 10874^T^

**DOI:** 10.1128/genomeA.01358-16

**Published:** 2016-12-08

**Authors:** Mina Ootsuka, Tomoyasu Nishizawa, Hiroyuki Ohta

**Affiliations:** a United Graduate School of Agricultural Science, Tokyo University of Agriculture and Technology, Fuchu-shi, Tokyo, Japan; b Ibaraki University College of Agriculture, Ami-machi, Ibaraki, Japan

## Abstract

*Sphingobium cloacae* JCM 10874^T^ can degrade phenolic endocrine-disrupting chemicals, nonylphenol, and octylphenol. Here, we report the complete genome sequence of the JCM 10874^T^ strain.

## GENOME ANNOUNCEMENT

Environmentally persistent alkylphenols, such as 4-nonylphenol (NP), are known to be estrogenic in animals (1). The chemicals are breakdown products of microbial degradation of man-made nonionic surfactants, namely, nonylphenol polyethoxylates (2). Tanghe et al. published the first report regarding the isolation of a bacterium able to degrade NP in 1999 (3). Following that study, two NP-degrading bacteria were isolated from sewage treatment plant wastewater and river sediment, which were later described as new species, *Sphingomonas* (*Sphingobium*) *cloacae* and *Sphingobium amiense* (4, 5). Further analysis of biodegradation activity revealed that *S. cloacae* S-3^T^ (JCM 10874^T^) displays a catabolic activity specific to branched alkylphenols, while *S. amiense* YT^T^ is a versatile organism capable of utilizing a wide range of phenolic compounds (6). Here, we investigated the genome of *S. cloacae* JCM 10874^T^ to obtain important information regarding the catabolic genes of NP-degrading *Sphingomonas* (*Sphingobium*) species.

Genomic DNA was isolated from *S. cloacae* JCM 10874^T^ and sequenced using PacBio RSII instruments. Reads were assembled using FALCON version 0.2.1 to produce seven contigs. The genome sequence was annotated using the Microbial Genome Annotation Pipeline (http://www.migap.org) and Rapid Annotations using Subsystems Technology version 2.0 (7), followed by manual annotation with the NCBI-nr databases using the BLASTP program (8). tRNAs were predicted using tRNAscan-SE (9).

The genome of *S. cloacae* JCM 10874^T^ consists of 3,767,292 bp in one circular chromosome (SCLO1), with a coverage of 322-fold and 64.6% G+C content, 3,293 coding sequences (CDSs), six rRNA operons, and 49 tRNA genes. There are also five circular plasmids. The plasmids are pSCLO2 (sequence coverage, 301-fold; total nucleotide sequences, 375,493 bp; G+C content, 64.9%; total number of CDSs, 334), pSCLO3 (sequence coverage, 340-fold; total nucleotide sequences, 151,712 bp; G+C content, 62.8%; total number of CDSs, 137), pSCLO4 (sequence coverage, 225-fold; total nucleotide sequences, 108,910 bp; G+C content, 63.7%; total number of CDSs, 92), pSCLO5 (sequence coverage, 85-fold; total nucleotide sequences, 57,701 bp; G+C content, 63.5%; total number of CDSs, 53), pSCLO7 (sequence coverage, 32-fold; total nucleotide sequences, 33,768 bp; G+C content, 62.9%; total number of CDSs, 33), and linear pSCLO6 (sequence coverage, 99-fold; total nucleotide sequences, 52,690 bp; G+C content, 62.4%; total number of CDSs, 51).

Two genes responsible for alkylphenol oxygenation at the initial step of degradation were detected: one copy of the octylphenol 4-monooxygenase gene (*opdA*) was present in pSCLO3, and one copy of the nonylphenol monooxygenase gene (*nmoA*) was present in pSCLO4. *opdA* shows an amino acid sequence identity of 99.1% with OpdA of *Sphingomonas* sp. TTNP3 (3, 10, 11), and *nmoA* shows 100.0% identity with NmoA of *Sphingomonas* sp. NP5 (12). Monooxygenase and dioxygenase genes related to cyclic hydrocarbon (cyclopentanone and hydroxyquinol) degradation were also found in the circular plasmids. A gene cluster (7,939 bp) that encodes a hydroquinone catabolic pathway, *hqdRAB-orf1-orf2-hqdCDEF*, was found in the linear plasmid. Amino acid sequence identities were 99.4% (HqdA) and 100% (HqdR, HqdB, HqdC, HqdD, HqdE, and HqdF) between *S. cloacae* JCM 10874^T^ and the well-characterized *Sphingomonas* sp. strain TTNP3 (3, 13, 14). These results suggest that the degradation of alkylphenols is conducted by the concerted action of different plasmid systems in the bacterium.

### Accession number(s).

The genome sequence of JCM 10874 was deposited in the DDBJ/EMBL/GenBank databases under the accession numbers AP017655 to AP017661.
